# Incidental ^68^ Ga -DOTATATE Uptake in Metastatic Clear Cell Renal Cell Carcinoma: A Case Report

**DOI:** 10.1055/s-0045-1813681

**Published:** 2025-11-23

**Authors:** Fatemeh Saboktakin, Saeed Farzanehfar, Nasim Vahidfar, Niloofar Tabatabaeian

**Affiliations:** 1 Department of Nuclear Medicine, Tehran University of Medical Sciences, Vali-Asr Hospital, Tehran, Iran

**Keywords:** gallium-68, neuroendocrine tumors, diagnostic, somatostatin receptor, renal cell carcinoma, DOTATATE

## Abstract

^68^ Ga-DOTATATE positron emission tomography/computed tomography (PET/CT) is a more accurate way to distinguish neuroendocrine tumors compared with conventional diagnostic methods and a standard impressive way to evaluate disease entities. This is based on ^68^ Ga-DOTATATE affinity to somatostatin receptors that are overexpressed in the majority of neuroendocrine tumors. In this case presentation, it is aimed to demonstrate the role of ^68^ Ga-DOTATATE PET/CT scan beyond the detection of neuroendocrine tumors.

A 59-year-old Iranian male patient with clear cell renal cell carcinoma who underwent nephrectomy 4 years ago has been referred for evaluation of a diagnosed painful abdominal mass by CT. ^68^ Ga-DOTATATE PET/CT scan showed a large, lobulated border ^68^ Ga-DOTATATE avid retroperitoneal tumoral mass in the region of pancreatic head and uncinate process, with fat plane obliteration with duodenum and inferior vena cava.

Conspicuous physiological uptake has been reported in the pancreatic head in 16 to 70% of ^68^ Ga-DOTATATE. Pathological results and immunohistochemistry evaluations confirmed the renal cell carcinoma based on metastases; so, the suspicious diagnosis of neuroendocrine tumor was ruled out.

Malignancies, a part of neuroendocrine tumors, may express somatostatin receptors and ^68^ Ga-DOTATATE or other octreotide derivatives based radiopharmaceuticals uptake appears.

## Introduction

Renal cell carcinoma (RCC) is one of the most frequent malignancies, with twice the prevalence in women than in men. 1 Prior to the introduction of targeted therapy, metastatic RCC was considered as a poor prognosis of disease, and treatment was imperfect; however, since 2005, systemic treatments for patients' survival have been improved. 2 3 Distinguishing RCC in adrenal glands and pancreas is complicated, since differentiation with primary tumors of these glands and thyroid lesions is difficult. 4 Also, adrenal and pancreatic lesions' identification is difficult because of anatomically located in retroperitoneal space. 4 While RCC expresses somatostatin receptors (SSTRs), radiopharmaceuticals with a capability of interaction with SSTRs would be efficient for diagnostic purpose of RCC, 5 which can be instructive for functional expression since histological evidences are poor. 6

SSTR-based 1,4,7,10-tetraazacyclododecane-1,4,7,10-tetraacetic acid (DOTA)–octreotate (DOTATATE, GaTate) positron emission tomography (PET)/computed tomography (CT), is an impressive imaging modality for diagnostic purpose of neuroendocrine tumors (NETs) with demonstrated superior specifications over the conventional methods. 7 The considerable point is that SSTR-2 is expressed by tumor cells in both primary tumor and metastases of RCC, that ^68^ Ga-DOTATOC PET/CT scan can specifically detect RCC metastases. 4 We report a patient with clear cell RCC whose diagnosis and management were guided by ^68^ Ga-DOTATATE PET/CT scan.

## Case Presentation

A 59-year-old man with a history of clear cell RCC, status postleft nephrectomy, was referred to the nuclear medicine department for evaluation of a newly developed painful mass in the uncinated process of the pancreas and multiple bilateral pulmonary nodules, identified on recent abdominal and chest CT scans.

As part of the diagnostic workup, a ^99m^ Tc-octreotide single-photon emission computed tomography (SPECT) scan was initially performed to assess for a possible NET. The scan demonstrated abnormal focal radiotracer uptake in the mid line of abdominal cavity corresponding to a lesion in the pancreatic head. No other abnormal ^99m^ Tc-octreotide-avid lesions were seen throughout the body.

**Fig. 1:**
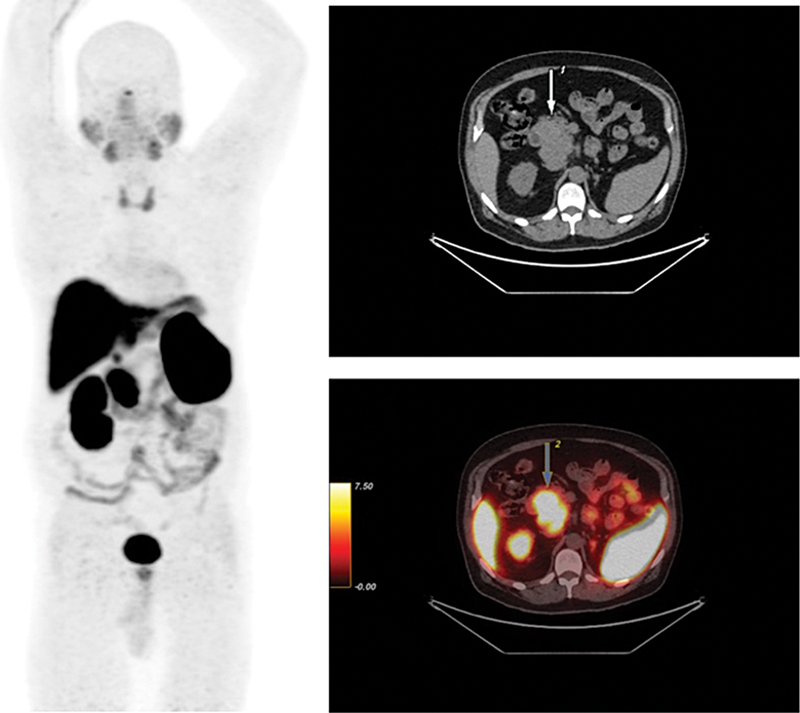
Maximum intensity projection, transaxial CT and fused PET/CT images demonstrated pathologically intense ^68^ Ga-DOTATATE uptake (SUV _max_ = 23.6) on the pancreatic mass in the head and uncinate process measuring 63 × 49 mm. Pathology and IHC evaluation confirmed the metastatic source of clear cell RCC. CT, computed tomography; IHC, immunohistochemical; PET, positron emission tomography; RCC, renal cell carcinoma; SUV, standardized uptake value.

**Fig. 2:**
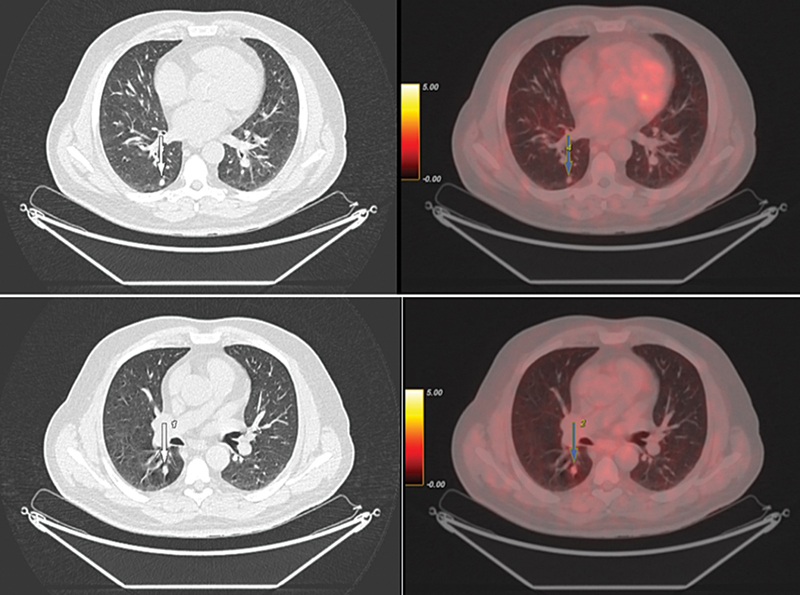
Transaxial CT and fused PET/CT images demonstrated lung nodules; some of them showed mild ^68^ Ga-DOTATATE uptake. These nodules were considered metastatic and patient referred for systemic therapy. CT, computed tomography; PET, positron emission tomography.

To further characterize the lesion, a ^68^ Ga-DOTATATE PET/CT scan was subsequently performed. This revealed a ^68^ Ga-DOTATATE-avid mass in the pancreatic head and uncinated process ( Fig. 1 ). Additionally, multiple bilateral pulmonary nodules were noted, a few of which demonstrated mild ^68^ Ga-DOTATATE uptake, raising suspicion for metastatic disease ( Fig. 2 ). The ^68^ Ga-DOTATATE PET/CT demonstrated higher lesion conspicuity and sensitivity compared with the prior ^99m^ Tc-octreotide SPECT scan.

For definitive diagnosis, the patient underwent endoscopic ultrasound-guided fine needle biopsy (EUS-FNB) of the pancreatic lesion. Histopathological analysis initially suggested a neuroendocrine-like tumor. However, immunohistochemical (IHC) profiling yielded the following results:

Cytokeratin: positive, synaptophysin: negative, chromogranin: negative, vimentin: positive, PAX8: positive, leukocyte common antigen: negative, MUC1: negative.

This IHC pattern was consistent with metastatic clear cell RCC, and not with a primary NET. Notably, somatostatin receptor (SSTR2) expression was not confirmed histologically, and its presence was inferred based on radiotracer uptake on functional imaging.

The patient was referred to oncologist for systemic therapy, including initiation of pembrolizumab.

## Discussion

SSTR-2 is expressed by tumor cells in primary clear cell RCC and corresponding metastases in thyroid, adrenal, and pancreatic glands. DOTATATE is therefore expected to bind to SSTR-2 in RCC metastases, enabling the use of ^68^ Ga-DOTATATE PET/CT as a functionally more specific method than fluorodeoxyglucose-PET and conventional CT. The case presented here highlights the importance of diagnostic differentiation of RCC from NETs based on ^68^ Ga-DOTATATE uptake. Basically this is about the physiological and pathological uptake of SSTR based radiopharmaceuticals by uncinated pancreas. The unique aspect of this report is about the EUS-FNB ratification of the outcome. There are some resembling clinical evidence, with the same approaches. Patel et al. reported a case with von Hippel–Lindau syndrome presented with ^68^ Ga-DOTATATE uptake in the pancreatic head, splenic hilar region, and multiple osseous sites, including the right lateral portion of the T9 vertebrae. 8 The final assessment was clarified by biopsy of the T9 lesion that approved metastatic RCC. 8 Vamadevan et al. also demonstrated uptake of ^68^ Ga- DOTATATE in a clear cell RCC. 9 This mimicking pattern of SSTR-based radiopharmaceuticals uptake by RCC has been reported frequently including ( ^68^ Ga-DOTATATE, ^68^ Ga-DOTANOC, ^68^ Ga-DOTATOC, and ^111^ In-octreotide). 10 11 12 13 14 15 16 Image-based assessments confirmed that in comparison to pathological uptake, physiological aspects of accumulation in the pancreatic head show lower PET standardized uptake value and is less focal in appearance. 17

In the case presented above, abdominal and chest CT scan also showed mild enhancements in the uncinated pancreas and lungs. While CT is the common cross-sectional imaging modality of NETs, findings of EUS-FNB could serve as complementary information of the final interpretation. In CT scan, a mass with distinct lobules and hyperenhancement in the head and uncinate of the pancreas was seen. The mentioned mass encased the distal periampullary area and caused dilation of the intrahepatic and extrahepatic bile ducts, so that the common bile duct was measured and the gallbladder was dilated. The right kidney showed compensatory hypertrophy and four simple cysts were seen in the kidney. Also, in limited cuts of chest, a nodule was seen at the base of the right lung as well as another nodule at the base of the left lung.

Highly suggested for RCC was reported by EUS-FNB. This can be mentioned that in cases where medical imaging or other noninvasive tests provide an unclear or ambiguous diagnosis, a biopsy is crucial for confirming the presence and nature of a suspected condition.

Finally, based on IHC assessments histological type of the mass was reported clear cell RCC. With this finding, it was revealed that ^68^ Ga-DOTATATE PET/CT scan can be emerged as a standard diagnostic way for evaluation of RCC dysfunctions with admissible outcome.

## Conclusion

It should be kept in mind that ^68^ Ga-DOTATATE and other SSTR base radiopharmaceuticals can be show uptake in some other malignancies apart from the NETs such as RCC, which mentioned in this case. The biopsy confirmation remains essential in ambiguous cases.
